# A rare case of hyperuricemia and acute kidney injury in a kidney transplant patient

**DOI:** 10.1007/s00467-025-06975-z

**Published:** 2025-10-13

**Authors:** Anna S. Nidhiry, Dechu Puliyanda, Cindy Blifeld, Helen Pizzo

**Affiliations:** 1 Department of Pediatrics, Seattle Children’s and University of Washington, Seattle, CA USA; 2 Department of Pediatrics, Cedars Sinai Guerin Children’s, 8700 Beverly Blvd, Los Angeles, CA 90048 USA; 3 Department of Pediatrics, Cottage Health, Santa Barbara, CA USA

**Keywords:** Uric acid, Cimetidine, Pediatric, Kidney transplant

## Abstract

Hyperuricemia is a common finding in kidney transplant recipients, often associated with calcineurin inhibitor or diuretic use, obesity, metabolic syndrome, dyslipidemia, high purine intake, and reduced allograft function. We report a unique case of hyperuricemia leading to acute kidney injury associated with the use of cimetidine for treatment of mosaic warts in a 12-year-old female pediatric kidney transplant patient. With the reduction in the patient’s serum uric acid levels, there was concurrent marked improvement in her eGFR. Subsequently, her uric acid remained low, suggesting the cessation of cimetidine maintained her baseline uric acid level. This case highlights the significance of monitoring uric acid levels post-transplant and the importance of attention to potential drug interactions.

## Case report

A 12-year-old female who received a deceased donor kidney transplant 9 years ago for stage 5 chronic kidney disease secondary to ischemic injury and renal vein thrombosis in the neonatal period presented with 4 days of fatigue, cough, and vomiting after starting a course of amoxicillin for right-sided acute otitis media a week prior. Her vital signs were normal, including a blood pressure of 99/72 mmHg. Body mass index was normal at 20.0 kg/m^2^ (73%). Laboratory workup was notable for creatinine of 12.56 mg/dL (eGFR 4.2 ml/min/1.73 m^2^), blood urea nitrogen of 92 mg/dL, uric acid of 27.2 mg/dL (reference mean for sex and age: 4.1 mg/dL, standard deviation 0.8 mg/dL) [1], and tacrolimus level of 20 ng/mL. Urinalysis showed no sediments or uric acid crystals; fractional excretion of uric acid was 12.8% (reference for age 6.47 ± 3.28%) [2]. Serum sodium was 137 mmol/L, potassium 3.5 mmol/L, chloride 102 mmol/L, and carbon dioxide 17 mmol/L. Two months prior, her lipid panel showed mildly elevated total cholesterol of 217 mg/dL (reference range < 200 mg/dL); high-density lipoprotein, low-density lipoprotein, and triglyceride levels were within the normal range. Her baseline serum creatinine was 1.53 mg/dL (eGFR was 34.7 ml/min/1.73 m^2^), and uric acid was 9.2 mg/dL. She completed her childhood vaccination series, including the human papillomavirus vaccine.

Patient’s immunosuppression consisted of tacrolimus (goal trough 5–7 ng/mL), mycophenolate mofetil (570 mg/m^2^/day), and prednisone; she is compliant in taking her medications, which also included enalapril for proteinuria, calcitriol, sodium bicarbonate, ergocalciferol, ferrous sulfate, erythropoietin, and fish oil for hypertriglyceridemia. For the past 2 months, she has also been taking cimetidine for treatment of mosaic warts that covered the toes and soles of her feet, which were resistant to prior treatment with cryotherapy, cantharidin, and imiquimod.

The patient was admitted for fluid resuscitation and kidney biopsy. On hospital day 1, home medications were continued, but the following medications were stopped: enalapril due to acute kidney injury, fish oil in anticipation of biopsy, and cimetidine due to potential association with hyperuricemia. One dose of tacrolimus was held due to the elevated level. Workup revealed no donor-specific antibodies, BK, CMV, or EBV viremia. The patient was started on allopurinol 50 mg every 3 days (renal dose) for severe hyperuricemia and on 1.5 times maintenance intravenous (IV) fluids.

**Fig. 1:**
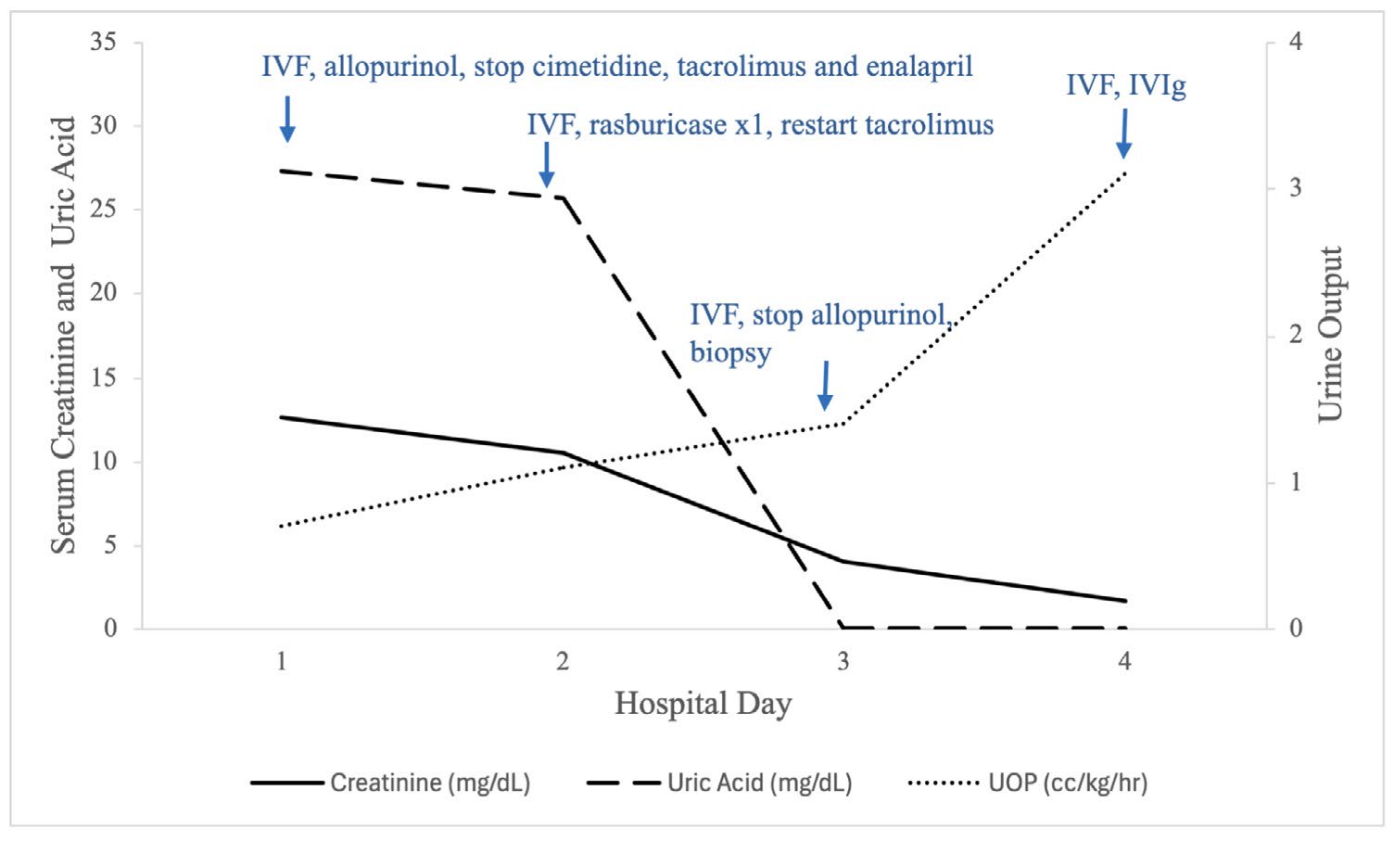
Change in serum creatinine, serum uric acid, and urine output in relation to medical interventions during hospitalization. IVF, intravenous fluids; IVIg, intravenous immunoglobulin; UOP, urine output

On hospital day 2, serum creatinine decreased to 10.46 mg/dL, and serum uric acid remained elevated at 25.7 mg/dL. A single dose of rasburicase 6 mg IV was given for persistent hyperuricemia, and a kidney transplant biopsy was performed. On hospital day 3, serum creatinine further decreased to 4.04 mg/dL, and serum uric acid was < 1.0 mg/dL, prompting discontinuation of allopurinol. Kidney biopsy showed evidence of antibody-mediated rejection; intravenous immunoglobulin 2 g/kg was initiated, followed by tocilizumab monthly following discharge. On hospital day 4, serum creatinine was 1.72 mg/dL, and uric acid remained < 1.0 mg/dL. UOP was 0.7 cc/kg/h over the first 12 h of admission, 1.1 cc/kg/h over the next 24 h, then 1.4 cc/kg/h the subsequent day, and 3.1 cc/kg/h the day of discharge (Fig. 1).

On follow-up 2 months later, uric acid was 7.7 mg/dL (for which allopurinol was initiated), and creatinine was 2.1 mg/dL. Her baseline uric acid level over the past 3 years fluctuated between 7.5 and 9.5 mg/dL without any hyperuricemia treatment before this admission. Lead and vitamin B12 levels were normal; peripheral smear, urinalysis, complete blood counts, and lactate dehydrogenase levels were unremarkable. Hematology-oncology workup for a possible lymphoproliferative or myeloproliferative process included a computed tomography scan of the chest/abdomen/pelvis without contrast to evaluate for post-transplant lymphoproliferative disorder, which was also normal.

## Discussion

Hyperuricemia is a common complication in kidney transplant recipients with a prevalence of 30 to 84% in the adult population and affecting 20% of pediatric kidney transplant patients [3]. Notably, in the pediatric population, uric acid levels change during development, gradually increasing with age and with sharper rates of rise in males compared to females [4]. Hyperuricemia is often associated with calcineurin inhibitor-based immunosuppression, such as cyclosporine and tacrolimus (though less pronounced effect with tacrolimus), due to alterations in tubular transport mechanisms leading to decreased urinary uric acid clearance [5]. Additional risk factors for hyperuricemia in kidney transplant recipients include obesity, metabolic syndrome, dyslipidemia, nonadherence to immunosuppressive therapy, diuretic use, and consumption of a high purine diet [6]. Hyperuricemia and kidney injury have a bidirectional relationship. Impaired kidney allograft function is a common cause of hyperuricemia in pediatric kidney transplant patients, as it often results in a decreased ability to filter and excrete uric acid, leading to its accumulation in the blood. Conversely, hyperuricemia post-transplant can cause kidney injury and contribute to eGFR decline over time [5]. Serum uric acid levels >10–15 mg/dL can lead to uric acid nephropathy and acute kidney injury through the deposition of uric acid in kidney tubules, resulting in inflammation and tissue damage [7]. Treatment often involves the use of a xanthine oxidase inhibitor (allopurinol), which decreases uric acid production, or a uric acid oxidase (rasburicase), which metabolizes uric acid to allantoin [4]. Therefore, the use of allopurinol in this patient was to prevent further uric acid production, and rasburicase was used to break down the existing uric acid.

Cimetidine is a histamine type-2 receptor antagonist indicated for peptic ulcer and gastroesophageal reflux disease and has also been effective in treating warts [8]. Through the blockade of histamine type-2 receptors, cimetidine acts as an immunomodulator to clear warts by stimulating Th1-type immune responses that result in increased production of IL-2, IL-12, TNF-α, and TNF-γ, enhancing cell-mediated immunity [8]. In healthy individuals, cimetidine is rarely a direct cause of hyperuricemia. Studies in adults with normal kidney function revealed that administration of cimetidine did not significantly increase serum uric acid concentration or decrease kidney clearance of uric acid [9]. However, cimetidine has been associated with increased uric acid levels in those with gout-like arthritis [10]. In kidney transplant patients, cimetidine has the potential to cause hyperuricemia through a variety of indirect mechanisms. One such mechanism is competition with uric acid for kidney excretion through organic anion transporters in the kidney, resulting in increased circulation of uric acid. Cimetidine can also interact with immunosuppressive drugs commonly used in kidney transplant patients that are associated with hyperuricemia, such as calcineurin inhibitors [5]. As a cytochrome P450 enzyme inhibitor, cimetidine can interfere with the metabolism of these drugs, also resulting in elevated serum levels of uric acid at higher doses [5].

## Conclusion

This case highlights the increased risk for hyperuricemia in kidney transplant patients due to both their underlying kidney dysfunction and the immunosuppressive medications required to maintain graft survival. It is important to note that while the patient had antibody-mediated rejection, her kidney function improved before treatment for rejection was initiated, and therefore, her acute kidney injury is unlikely to be solely secondary to the acute rejection. Although it is difficult to determine whether the improvement in eGFR for this patient was due to fluid resuscitation, decrease in uric acid levels, decrease in tacrolimus level, or discontinuation of angiotensin-converting enzyme inhibitor, medications such as cimetidine can significantly exacerbate hyperuricemia by interfering with kidney excretion mechanisms and compromising kidney function. Although there were no histologic findings of uric acid nephropathy on biopsy, the patient’s creatinine did not markedly improve (despite aggressive hydration) until rasburicase was administered resulting in a drastic decline in uric acid levels. Given that transplant recipients often have some degree of hyperuricemia at baseline, it is essential to closely monitor serum uric acid levels and adjust treatment regimens accordingly to prevent complications such as acute kidney injury.

## Summary

### What is new?

- This case demonstrates the importance of continually assessing the potential impact of all prescribed medications on uric acid metabolism in the transplant population to avoid amplifying their risk for hyperuricemia.

## Data Availability

The data that support the findings of this study are available from the corresponding author upon reasonable request.

## Abbreviations

BK: BK polyoma virus
CMV: Cytomegalovirus
EBV: Epstein-Barr virus
eGFR: Estimated glomerular filtration rate
IV: Intravenous

## References

[1] Kubota M, Nagai A, Tang L, Tokuda M (2011) Investigation on hyperuricemia in children with obesity or various pediatric disorders. Nucleosides Nucleotides Nucleic Acids 30:1051–1059. 10.1080/15257770.2011.597370 22132956 10.1080/15257770.2011.597370

[2] Choi H, Namgoong M (2019) Does fractional excretion of uric acid correlate with urine uric acid creatinine ratio in spot urine? Nephrol Dial Transplant 34(Suppl 1): gfz106.FP828. 10.1093/ndt/gfz106.FP828

[3] Uslu Gokceoglu A, Akman S, Koyun M, Comak E, Dogan CS, Akbas H et al (2013) Hyperuricemia in pediatric renal transplant recipients. Exp Clin Transplant 11:489–493. 10.6002/ect.2013.0012 24344940 10.6002/ect.2013.0012

[4] Kubota M (2019) Hyperuricemia in children and adolescents: present knowledge and future directions. J Nutr Metab 2019:3480718. 10.1155/2019/3480718 31192008 10.1155/2019/3480718 PMC6525889

[5] Zi X, Zhang X, Hao C, Wang Z (2022) Risk factors and management of hyperuricemia after renal transplantation. Front Surg 9:956213. 10.3389/fsurg.2022.956213 36760666 10.3389/fsurg.2022.956213 PMC9904410

[6] Eyupoglu S, Eyupoglu D, Kendi-Celebi Z, Akturk S, Tuzuner A, Keven K et al (2017) Risk factors of hyperuricemia after renal transplantation and its long-term effects on graft functions. Transplant Proc 49:505–508. 10.1016/j.transproceed.2017.01.006 28340822 10.1016/j.transproceed.2017.01.006

[7] Fathallah-Shaykh SA, Cramer MT (2014) Uric acid and the kidney. Pediatr Nephrol 29:999–1008. 10.1007/s00467-013-2549-x 23824181 10.1007/s00467-013-2549-x

[8] Das BB, Anton K, Soares N, Riojas S, McDermott J, Knox L et al (2018) Cimetidine: a safe treatment option for cutaneous warts in pediatric heart transplant recipients. Med Sci 6:30. 10.3390/medsci6020030 10.3390/medsci6020030 PMC6024571 29642499

[9] Berardi RR, Hyneck ML, Cohen IA, Cornish LA, Achem SR (1984) Effect of cimetidine on serum uric acid concentration. Clin Pharm 3:56–59 6697675

[10] Einarson TR, Turchet EN, Goldstein JE, MacNay KR (1985) Gout-like arthritis following cimetidine and ranitidine. Drug Intell Clin Pharm 19:201–202. 10.1177/106002808501900306 3979260 10.1177/106002808501900306

